# Neural correlates of the non-optimal price: an MEG/EEG study

**DOI:** 10.3389/fnhum.2025.1470662

**Published:** 2025-01-28

**Authors:** Aleksei Gorin, Elizaveta Kuznetsova, Andrew Kislov, Egor Levchenko, Vasily Klucharev, Victoria Moiseeva, Anna Yurchenko, Alexander Luzhin, Natalia Galkina, Anna N. Shestakova

**Affiliations:** ^1^National Research University Higher School of Economics, Moscow, Russia; ^2^Faculty of Educational Sciences, University of Helsinki, Helsinki, Uusimaa, Finland; ^3^Faculty of Humanities, Center for Language and Brain, National Research University Higher School of Economics, Moscow, Moscow Oblast, Russia; ^4^JSC Neurotrend, Moscow, Russia

**Keywords:** EEG, MEG, N400, neuromarketing, neuroeconomics, consumer neuroscience, pricing

## Abstract

**Introduction:**

Setting the right price is crucial for effectively positioning products in the market. Conversely, setting a “non-optimal price”—one that is perceived as much lower or higher than the product's true market value—can negatively influence consumer decisions and business results.

**Methods:**

We conducted two electroencephalography (EEG) studies and one magnetoencephalography (MEG) study to investigate brain mechanisms underlying the perception of prices during a price judgment task. In each trial, participants were exposed to a mobile phone image (iPhone, Nokia, or Xiaomi) followed by a price, and instructed to judge whether the price was high or low based on a target word (“cheap” or “expensive”).

**Results:**

In both EEG experiments, we found a strong N400-like response to the incongruent target words following prices that substantially differed from the real market value of the mobile phone. The MEG experiment extended these findings by localizing the brain source of the price-related, M400-like response, the magnetic counterpart to the N400 component, in the ventromedial prefrontal cortex (vmPFC) and anterior cingulate cortex (ACC) implicated in value-based and reward-based learning, respectively. Our results demonstrate that both the brain sources and the timing of the price-related M400 response differed from those of the standard M400 evoked by semantically incongruent words.

**Discussion:**

Overall, our results suggest that the N400-like response can serve as a neural marker of price-product incongruity, with potential applications in consumer research.

## Introduction

Setting an optimal price—price point—is essential for maximizing business outcomes. There are different pricing strategies, which vary from a competitive analysis and managerial subjective opinion to an econometric-based market analysis (Dolgui and Proth, 2010). Setting a non-optimal price for a product could immediately decrease revenue or confuse customers who may misunderstand the product positioning. In the current study, we searched for neural correlates of price points that were not optimal (too low or too high) in the electro- and magneto-physiological brain activity of participants observing products and their prices.

Fundamental research in economics and marketing suggests that a customer's willingness to purchase a product largely depends on their perceived subjective value of the item. Economic methods for estimating price perception include various tools and techniques from both theoretical and experimental economics. These methods aim to understand how consumers evaluate the value of goods and services. For instance, microeconomic models often explore the concept of utility maximization, where individuals allocate their resources to derive the greatest satisfaction. Experimental methods, such as auctions and surveys, provide empirical data to support these theories. Such an effective, or optimal price, could be defined in several ways. One of the most promising approaches is setting the price to match the customers' maximum willingness to pay (WTP; Liozu et al., 2012). For example, WTP can be measured using a Becker–DeGroot–Marshak auction (Becker et al., 1964), where participants are offered to bid on a selected item within a given range. This view is consistent with the microeconomic term of a “customer's reservation price”—the price at which the consumer is indifferent to buying and not buying a product (Moorthy et al., 1997). Another widely used method for estimating consumer price perception is the Price Sensitivity Meter (European Society for Opinion and Marketing Research., 1976). This technique involves surveying consumers to identify the price points at which they perceive a product to be “too cheap,” “inexpensive,” “expensive,” or “too expensive.” By analyzing these responses, businesses can establish the acceptable price range for the product and determine the optimal price point that maximizes both demand and revenue. If the product is priced below this optimal point, more consumers may perceive it as “too cheap,” which can diminish its perceived value and deter purchases. Therefore, this optimal price point acts as the lower bound of acceptable prices. However, Ariely and Berns (2010) highlighted that a potential weakness of many traditional measurements of consumer price perception lies in the reliance on stated consumers' preferences, which can significantly differ from their real preferences. To avoid subjective biases, neuroscientific methods may offer more direct neural correlates of consumer price perception using neuroimaging methods.

During recent decades, value-based decisions to buy a product have been actively investigated using functional magnetic resonance imaging (fMRI; Plassmann and Karmarkar, 2015; Venkatraman et al., 2015; Plassmann et al., 2012) and electroencephalography (EEG; Ma et al., 2007, 2014, 2020; Ramsøy et al., 2018; Wang et al., 2012; Dmochowski et al., 2014; Falk et al., 2012; Kislov et al., 2023; Knutson and Genevsky, 2018; Raab et al., 2011; for a review, see Bazzani et al., 2020). Using fMRI, Hilke Plassman showed that the medial orbitofrontal cortex (mOFC) as well as dorsolateral prefrontal cortex (dlPFC) play a pivotal role in determining individuals' WTP for products. By encoding the subjective value of items based on various attributes and contextual factors, the OFC and dlPFC facilitate informed economic decisions.

Knutson et al. (2007) found that activation in regions associated with anticipating gain, such as the nucleus accumbens (NAcc), correlated with product preference, while activation in regions associated with anticipating loss, such as the insula, correlated with excessive prices. Further, activation in a region implicated in integrating gains and losses, the medial prefrontal cortex (mPFC), correlated with reduced prices (conversely, excessive prices deactivated mPFC). Research by Basten et al. (2010) found that the vmPFC, in particular, plays the role of an integrator of neural signals related to both benefits and costs. Specifically, the vmPFC combines these benefit and cost signals into a unified, difference-based representation of subjective value. Some studies have also shown that the vmPFC plays a key function in brand preferences and price-based decision-making (Bush et al., 2002; Koenigs and Tranel, 2008; Paulus and Frank, 2003; Plassmann et al., 2007; Wallis and Miller, 2003). Research by Rushworth et al. (2004) demonstrated the involvement of the anterior cingulate cortex (ACC) in evaluating rewards and costs, showing that it integrates information from various sources to guide value-based choices. ACC uses the history of reinforcement to predict the likely outcomes of different choices, helping to anticipate which actions may lead to rewards or avoid punishments based on what has happened before.

Overall, value-based decision-making is complex cognitive process that engages multiple interconnected brain regions, each contributing uniquely to different aspects of decision-making and not limited to critical regions mentioned above.

While fMRI remains costly and impracticable in field research, portable and less costly EEG has become a more and more popular instrument in consumer neuroscience (Ma et al., 2007, 2014, 2020; Ramsøy et al., 2018; Wang et al., 2012; for a review, see Bazzani et al., 2020; Boksem and Smidts, 2015). For example, applying the power spectral analysis to EEG data, Ramsøy et al. (2018) estimated the prefrontal asymmetry index and found that among other brain frequencies, the prefrontal gamma asymmetry was most strongly related to individuals' WTP responses. Moreover, the data analysis revealed that as the decision point approached, the relationship between WTP and gamma asymmetry became increasingly stronger.

Over the past 15 years, the event-related potential (ERP) technique has gained popularity in consumer neuroscience (e.g., Camarrone and Van Hulle, 2019; Fudali-Czyż et al., 2016; Harris et al., 2019; Hsu and Chen, 2020; Ma et al., 2014, 2020; Jin et al., 2015). ERP is a neuroimaging method used to measure the brain's electrical response to specific sensory, cognitive, or motor events. Derived from EEG, ERPs involve averaging the electrical brain signals time-locked to the occurrence of stimuli or actions. This averaging process enhances the phase locked signals related to the event while minimizing unrelated brain activity and noise (Luck and Kappenman, 2012). Gajewski et al. (2016) used an ERP approach to seek neuromarkers of willingness-to-buy (analogous to WTP) of electronic devices for prices that deviated from the average market price. In the trials in which the participants paid for an overpriced item as well as if they refused to buy a relatively cheap item, a conflict-related frontocentral N2 component (a negative deflection peaking at ~200 ms; it has been shown that the amplitude of the frontal N2 is associated with cognitive control and conflict detection; Jin et al., 2017) has been enhanced compared to the trials in which a product was purchased for the average price. The N2 enhancement was accompanied by an increase of the attention-related P3 component in the trials in which an overpriced product was purchased (Polich, 2007). The P3 is a positive ERP component that peaks at ~300 ms. It can be detected across a wide range of tasks related to attention, including oddball detection or stimulus recognition (Polich, 2007). Another EEG study demonstrated that the falsified price triggered an enhancement of the fronto-central N2 component followed by a decrease in the late positive potential (LPP; Fu et al., 2019; Münte et al., 2000). The LPP arises around 400 milliseconds after stimulus onset, lasting several hundred milliseconds, with peak amplitude at centro-parietal sites ~600 ms post stimulus. It reflects attentional and cognitive engagement, particularly in the evaluative categorization of motivationally relevant stimuli (Ito and Cacioppo, 2000). Neuromarketing research highlights its role in decision-making, showing that positively evaluated stimuli elicit enhanced LPP responses (Chen et al., 2010; Wang et al., 2016; Jin et al., 2017). This suggests the LPP integrates affective and cognitive appraisals during the late stages of decisions, making it a valuable marker for studying consumer behavior and purchase intent.

Importantly, the LPP is frequently preceded by the N400 component (Herring et al., 2011)—a negative deflection that peaks at ~400–500 ms after the presentation of a meaningful stimulus. The N400 is often associated with semantic incongruency, and it can be elicited by most meaningful stimuli, including isolated words, pseudowords, and pictures. The N400 component increases with increasing cognitive effort involved in semantically integrating the stimulus in its context and is attenuated by an item's predictability within a given context (Brown and Hagoort, 1993; Chwilla et al., 1995; Halgren et al., 2002; Kutas and Federmeier, 2011; Kutas and Hillyard, 1980). Various neuroimaging studies demonstrated a link between the N400 and the semantic processing of various types of stimuli (Lau et al., 2008), including semantic violations of brand association (Camarrone and Van Hulle, 2019; Dini et al., 2022; Ma et al., 2007, 2020; Shang et al., 2018; Wang et al., 2012; Gorin et al., 2022). In our study, in contrast to the previous studies, we focus on the sensitivity of the N400 component to the perceived violation of the optimal price point.

To the best of our knowledge, no magnetoencephalographic (MEG) studies explored consumers' price perception. However, Halgren et al. (2002) recorded the magnetic equivalent of the N400 response, known as classical semantic M400, as participants read sentences ending with semantically congruous or incongruous words. The authors demonstrated that less expected terminal words evoked a larger magnetic field over the left hemisphere, peaking at ~450 ms. A source modeling technique mapped the M400 generator in the left superior temporal sulcus (STS). High temporal and spatial resolution of MEG fuels the scientific interest in investigating the MEG correlates of product–price association, how individuals perceive and evaluate the relationship between products and their prices, and in comparing them with the neural signatures of the classical semantic network.

In the current study, we hypothesized that the N400 can serve as a neural signature of the product-price association: exposure to a price that is significantly lower or higher than the optimal price point should lead to a stronger N400 compared to exposure to a price that is near the optimal price point. To test this hypothesis, we constructed a simple *price judgment* task, in which the participants were asked if a price was too high or too low for a given model of a mobile phone. During the price judgment task, participants were exposed to an image of a mobile phone followed by a price and a target word (“expensive” or “cheap”). Importantly, some of the prices matched the real market price (RMP) of the mobile phone (congruent price), while some prices were above or below the real market price (incongruent price). To ensure that the participants were familiar with the product category, we used mobile phones that represent different price segments. To check our hypothesis, during EEG Experiment 1, we recorded brain responses to the target words following the price of the premium smartphone (iPhone) or low-cost (Nokia) mobile devices (for mobile phone model details, see the Methods section). During EEG Experiment 2, we used the same setup but replaced the well-known, low-cost (Nokia) mobile phone with a more expensive smartphone (Xiaomi) that was relatively new to the local market at that time. This setup allowed us to check whether the results were reproducible and could be generalized to the different segments of products.

In our final study, MEG Experiment 3, we used MEG to pinpoint the brain regions responsible for the price-related neural responses observed in our EEG studies and compared them with those involved in the traditional semantic network. To distinguish the price-related M400 occurring in the price judgment task with the classical semantic M400 observed in semantic tasks, we introduced a control condition that elicited the classical M400 response to semantically incongruent words. Thus, we aimed to determine whether our price judgment task activates a neural network similar to the one that generates the classical M400 response. We hypothesized that the price-related neural networks would mirror those involved in generating the traditional M400.

Altogether, we conducted three separate experiments including two exploratory EEG studies (Experiment 1 and Experiment 2) and one hypothesis-testing MEG experiment (Experiment 3), using similar basic experimental designs to (1) test whether we can record N400 (M400) responses as an index of the incongruent price point and (2) determine the extent to which the brain source of the standard semantic N400 (M400) overlaps with the brain source of the incongruent-price-related N400 (M400).

## Methods

### Participants

#### EEG Experiment 1

For the first EEG experiment, we recruited 24 right-handed participants. Three participants were excluded from the data analysis because of an extensively noisy EEG signal. Thus, the final data set included 21 participants (12 females, 18–29 years old, and median age = 21).

#### EEG Experiment 2

For the second EEG study, we recruited 22 participants. Three participants were excluded from the data analysis due to an extensively noisy EEG signal. Thus, the final data set included 19 participants (11 females, 18–27 years old, and median age = 21).

#### MEG experiment

For the final experiment, we recruited 25 right-handed participants (17 females, 23–28 years old, and mean age = 25).

We recruited the respondents for each experiment using the same approach and targeted the same auditory to reduce effects of age, income, etc. on the data which allowed us to compare the results across the research. Respondents entered the study once, meaning that in total we collected data from 65 different persons. All participants had normal or corrected-to-normal vision and no history of head injuries, language disorders or mental illness. Also, none of the participants of the MEG Experiment had metal implants that could distort the MEG signal. Handedness was assessed with the Edinburgh Handedness Inventory (Oldfield, 1971). The respondents declared middle or upper-middle income status. All participants provided written informed consent and were naïve to the main purpose of the study. The study protocol was approved by the local ethics committee of the University (with details of the specific institution to be disclosed upon acceptance of the paper). At the beginning of the experiment, each participant was informed about the experimental procedure and EEG/MEG method.

### Stimuli and procedure

#### Price judgment task (EEG Experiment 1, EEG Experiment 2, and MEG Experiment 3)

##### Trial structure

**Figure 1:**
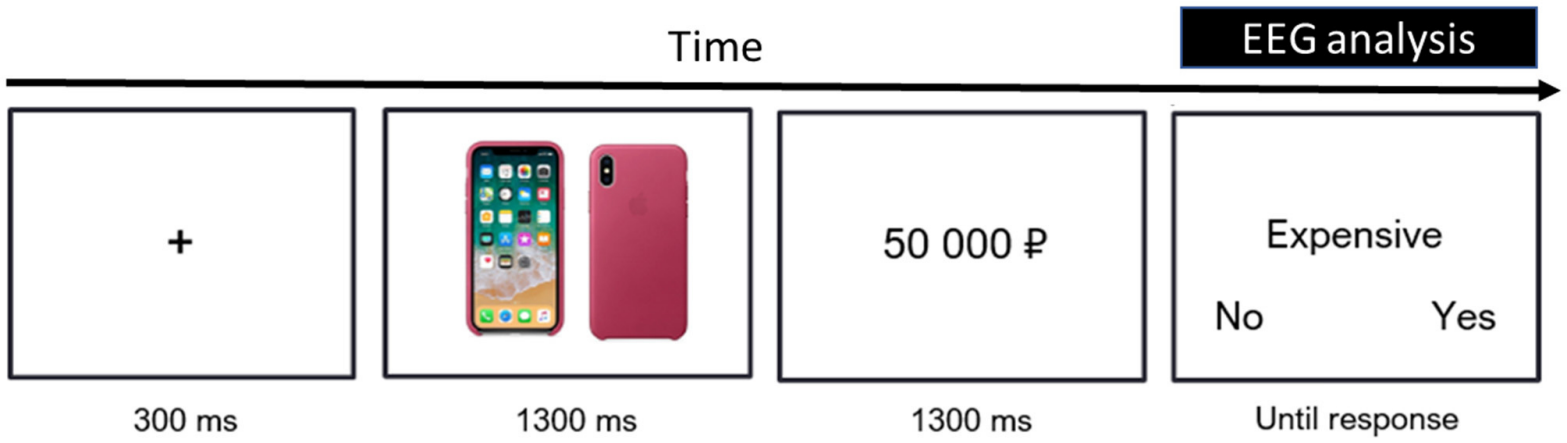
Trial structure of the price judgment task. Participants were asked to agree or disagree with a statement labeling the phone's price as “cheap” or “expensive.” ERPs and event-related fields (ERFs) were locked to the onset of the target word (marked by a black rectangle).

Figure 1 shows a sample trial of the experiment, in which the participants were exposed to a picture of a mobile phone white background followed by its hypothetical price and a target word (“expensive” or “cheap”). During the last response stage, the participants were asked to indicate whether the target word was adequate for the price and to press the corresponding button (Figure 1).

##### Stimuli

In all experiments, the participants were exposed to photos of two models of mobile phones (Stimulus #1 and Stimulus #2). In EEG Experiment 1, we used photos of the Apple iPhone XS smartphone as Stimulus #1 and photos of the Nokia 105 mobile phone as Stimulus #2. In EEG Experiment 2, we used photos of the Apple iPhone XS smartphone as Stimulus #1 and photos of the Xiaomi Mi A2 as Stimulus #2. In MEG experiment 3, we used photos of the same pair of mobile phones shown in EEG Experiment 1.

Each mobile phone was presented in different colors and at different angles to reduce the monotonicity of the visual stimulation. We used two angles and four colors (8 pictures) for each phone; the pictures were evenly distributed across the trials being randomly assigned to the prices within the price range (5 presentations per price range per target word). The order of the prices, target words and stimuli was counterbalanced.

In EEG Experiments 1 and EEG Experiment 2, participants were exposed to products and a wide range of prices (980–11,000 monetary units that had been grouped into five price ranges, 40 prices per range. Collectively, the EEG experiments consisted of 800 randomized trials (40 trials × 5 ranges × 2 products × 2 target words). The price ranges, which approximately corresponded with real market prices, were classified as matched to the “real market value” (RMV). Price ranges, which were higher than the real market price, were classified as prices above market value (AMV). The price ranges, which were lower than the real market price, were classified as “below market value” (BMV). Table 1 summarizes prices ranges that were used in three experiments.

**Table 1:** Prices used in the experiments.

**Price range**	**Monetary units**	**Price range relative to the market price for Nokia 105**	**Price range relative to the market price for Apple iPhone XS**	**Price range relative to the market price for Xiaomi**	**Experiments where a given price range was used**
1	980–2,200	Below market value	Below market value	Below market value	EEG Experiments 1 and 2, MEG Experiment 3
2	3,000–6,900	**Real market value**	Below market value	Below market value	EEG Experiments 1 and 2
3	7,000–23,000	Above market value	Below market value	**Real market value**	EEG Experiments 1 and 2
4	26,000–66,000	Above market value	**Real market value**	Above market value	EEG Experiments 1 and 2
5	70,000–110,000	Above market value	Above market value	Above market value	EEG Experiments 1 and 2, MEG Experiment 3

In MEG experiment 3, we used only marginal price ranges (lowest and highest prices—price range 1 and 5, Table 1) and focused on the iPhone mobile phone, therefore collecting 160 trials. This decision was made to reduce the experiment duration (using a full-length price judgment task and semantic task would extend the recording up to 3 h with preparation). Other price ranges and trials, which presented photos of the Nokia mobile phone, were used as filler probes: four trials per price range, and 32 filler trials in total. These probes were not analyzed further. Similar to the EEG experiments at the end of each trial, the target word “cheap” or “expensive” was presented.

For detailed information about product–price combinations and price ranges, see Supplementary Table 1.

To reduce the monotony of the EEG experiments, we added 20 filler trials, in which the participants were presented with curious historical facts from telecommunication history (for example, “usually, people unlock their phones 110 times per day”). Participants could agree or disagree with the statement presented on the screen using the keyboard. These trials were not included in analysis. The routine of EEG Experiment 2 was the same as that in EEG Experiment 1, but images of the Nokia cell phone were replaced by images of the Xiaomi smartphone.

#### Control semantic N400/M400 task (MEG Experiment 3)

To localize the standard N400/M400 response to the semantically incongruent words, we used the classic semantic N400 task. The stimuli consisted of 40 short sentences containing congruent and incongruent (critical) words for the third position in the sentence. Words were manipulated following the balanced design proposed by Steinhauer and Drury (2012). All critical words were two syllables in length and were presented in the native language.

**Figure 2:**
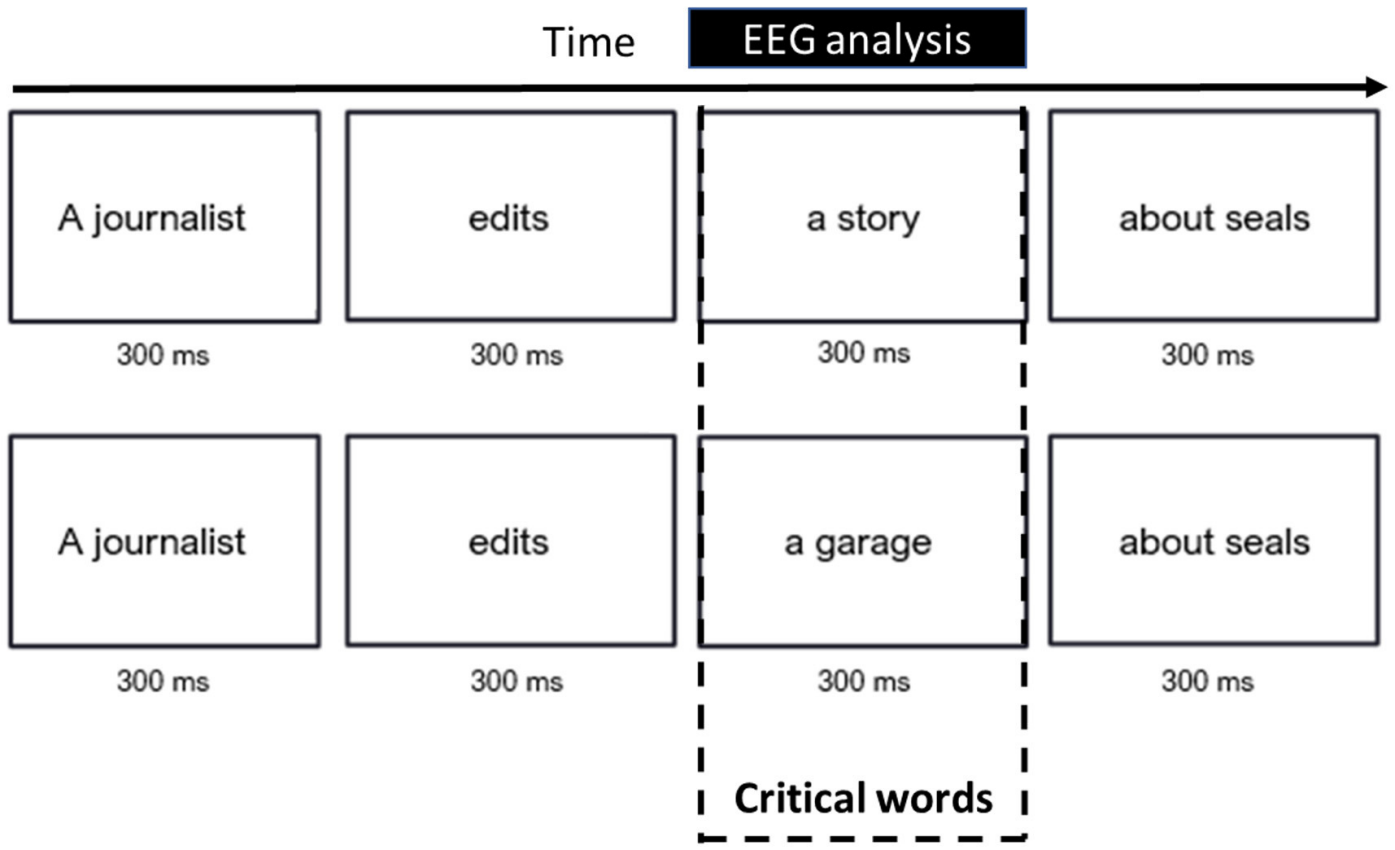
Trial structure of the control semantic N400/M400 task (MEG Experiment 3). Top: semantically *congruent trial*; bottom: semantically *incongruent trial* (translated into English). The MEG analysis was locked to the onset of the critical word (marked by a rectangle).

The sentences were presented word by word in black on a white background with a 300 ms interstimulus interval (see Figure 2) and an intertrial interval of 0.1–0.3 s. To maintain the participants' attention during the task, they were randomly asked (33% of the sentences) to make a judgment about the meaning of the sentence. Evoked activity was locked to the onset of the critical word.

In the MEG study, to counterbalance the experimental tasks, 12 participants started the experiment with the semantic N400/M400 task, and 13 participants started with the price judgment task. The MEG study lasted ~60 min, including preparation for MEG recordings and practice trials. A short break of 3 min separated the tasks.

#### EEG recording and analysis—EEG Experiments 1 and 2

We recorded EEG with the Brain Products Actichamp system using 60 active electrodes positioned according to the extended version of the 10–20 system with impedance stated on the level, < 5 kΩ prior to the recordings. EEG channels were referenced against an averaged signal from two mastoid electrodes. An electrooculogram (EOG) was recorded from electrodes placed below the right eye and over the left outer canthi. The online 50 Hz notch filter was applied during the data acquisition. The ground electrode was placed on the Fpz site.

Data analysis of EEG was performed using the Brainstorm toolbox (Tadel et al., 2011). Raw EEG signals were visually inspected for artifacts, and noisy segments were removed from further analysis. EEG data were offline filtered in the 1–40 Hz range. To correct eye movement artifacts, we used independent components analysis (ICA). The eye movement components were removed according to their topography. After preprocessing, we imported-−200 to 800 ms baseline-corrected (−100 to −2 ms) epochs locked to the target word onsets.

We examined an N400 locked to the onset of the target word using a series of false discovery rate (FDR)-corrected paired permutation tests: for each product we ran five tests, one per price range, comparing ERPs to “cheap” vs. ERPs to “expensive” target words. Our analysis focused on the time window from 300 to 500 ms post-stimulus based on previous literature on the N400 component (Lau et al., 2008). Since we had an a priori hypothesis that the N400 magnitude should vary as a function of the price incongruency, we focused our analysis on a cluster of six electrodes demonstrating the most negative deflection in the incongruent AMV trials, in which the experimental price was much higher than the actual product price (5th range for Nokia in Experiment 1; see the topography in the Supplementary Figure 1).

Importantly, depending on the price range (BMV/RMV/AMV) and the product, the (in)congruency of the target words (“cheap”/”expensive”) reversed in the trials with RMV. More precisely, the target word “cheap” was congruent for the BMV prices and required a “yes” response, but it was incongruent for the AMV prices and required a “no” response. Simultaneously, the target word “expensive” was incongruent for the BMV prices and required a “no” response, but it was congruent for the AMV prices and required a “yes” response. Overall, we compared the N400-responses averaged across the cluster of electrodes and price ranges within the 300–500 ms time window with a series of FDR-corrected permutation tests (ERPs to “cheap” versus ERPs to “expensive” target words).

#### MEG recording and data analysis

Magnetic fields were measured using a 306-channel whole-head Elekta Neuromag VectorView MEG scanner (Elekta AB, Sweden), comprising 102 magnetometers and 204 planar gradiometers, with a sampling rate of 1,000 Hz. Data were continuously recorded with a band-pass filter applied between 0.1 and 333 Hz. To reduce external noise, the temporally extended source signal separation (tSSS) method was applied post-acquisition (Taulu and Hari, 2009). Head movements were corrected to default head coordinates using signals from four head position indicator (HPI) coils positioned at F3, F4, and bilaterally on the mastoids. All procedures utilized Elekta Neuromag's MaxFilter software (Wilson et al., 2007). For precise co-registration of MEG and MRI data, a Polhemus Fastrak motion tracker (polhemus.com) was used to digitize three anatomical landmarks (the nasion and two preauricular points), HPI coil locations, and 100 additional scalp points.

Data analysis was performed using the MATLAB-based Brainstorm toolbox (Tadel et al., 2011). Prior to the analysis, the raw data were manually inspected for unspecific artifacts, and corresponding data were removed from further analysis. Next, the data were band-pass filtered (1–50 Hz) and sent to ICA to correct for cardiac and eye-movement artifacts. We performed separate ICA for the magnetometers and gradiometers. For the event-related fields (ERF) analysis, continuous MEG recordings were divided into epochs (−200 to 800 ms) locked to the onset of the target word (price judgment task) or critical word (semantic task N400/M400). ERFs were computed by averaging the epochs for each trial type for each participant separately. Two participants were removed from the further MEG analysis because of noisy data.

Since the goal of MEG Experiment 3 was to compare the price-related N400/M400 with the standard semantic N400/M400, in this version of the price judgment task, we used only marginal price ranges to statistically compare evoked responses to *congruent* target words [“cheap” for the BMV (range 1) and “expensive” for the AMV (range 5)] and *incongruent* target words [“expensive” for the BMV (range 1) and “cheap” for the AMV (range 5)].

#### Sensor space MEG analysis

We analyzed the evoked activity in the typical N400 time window (300–500 ms; Frank et al., 2015, p. 20; Kutas and Federmeier, 2011; Payne et al., 2015) with a series of paired permutation tests with the number of randomisations set to 1,000. We statistically compared ERFs in *incongruent* and *congruent* trials collected in both the price judgment task and the semantic N400/M400 task. The results were corrected for multiple comparisons using the FDR approach, as implemented in the Brainstorm interface. During further analysis, we separated all price judgment task trials into *range 1* and *range 5* trials to compare the ERFs in different price contexts.

#### MEG source space analysis

Individual T1-weighted MRI scans were acquired for all participants using a 1.5 T Siemens scanner. Three-dimensional brain models were then constructed for each subject using the FreeSurfer software toolbox (Fischl, 2012) and imported into the Brainstorm workspace. Forward solutions for the individual head models were computed using the overlapping spheres method.

We used the Brainstorm implementation of the sLORETA (Pascual-Marqui, 2002) estimation algorithm with a constrained orientation to obtain the cortical current density distribution underlying the observed ERFs. The absolute values of activation were then computed for each vertex and projected from the individual head models onto the default anatomy model (6,000 vertices) by using the iterative closest neighbor search algorithm, as implemented in the Brainstorm software.

To compare the responses between *incongruent* trials and *congruent* trials (Oostenveld et al., 2011), we used the Brainstorm interface of the Fieldtrip toolbox to perform a series of paired spatiotemporal cluster-corrected permutation tests (the cluster inclusion threshold was set to *p* < 0.01 with 1,000 permutations) over a continuous M400-related time window (300–500 ms). The cluster *p*-values were defined as the probability of observing the cluster with the higher mass separately for the positive and negative clusters.

## Results

### EEG Experiment 1

**Figure 3:**
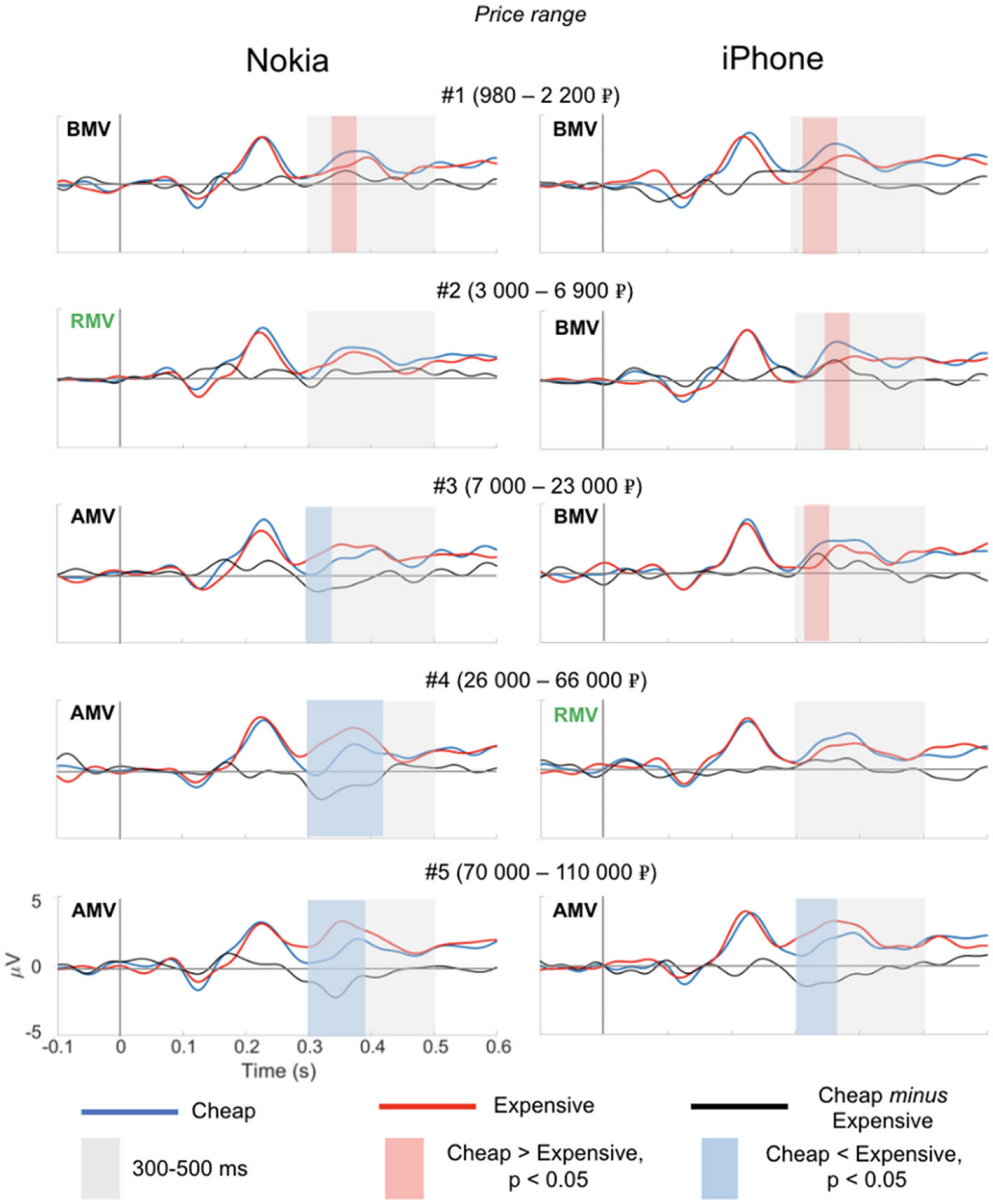
Event-related potentials to the target words “cheap” (blue curve) and “expensive” (red curve) in EEG Experiment 1. Across the price ranges, the N400 component reverses polarity: in the context of relatively cheap prices (BPV price ranges), the N400 was evoked by the target word “expensive.” However, in the context of relatively high prices (APV price ranges), the N400 was evoked by the target word “cheap.”

In the first study, we discovered that the magnitude of the N400 response gradually changed in line with the price ranges (Figure 3). As expected, we observed an inversion of the polarity of the N400 component: for BPV price ranges, we observed a positive N400 (ERP to the target word “cheap” *minus* the ERP to the target word “expensive”). For the APV price ranges, we observed a negative N400. Thus, for both mobile phones in the context of relatively cheap prices (BPV), the N400-like deflection was evoked by the target word “expensive.” However, in the context of relatively high prices (APV), the N400-like deflection was evoked by the target word “cheap.”

The statistical analysis (*p* < 0.05, FDR-corrected) confirmed a significant *positive* deflection of the difference wave (ERP to the target word “cheap” *minus* the ERP to the target word “expensive”) in the trial with BMV price ranges, both for the iPhone (ranges 1, 2, and 3) and Nokia mobile phone (range 1). A significant *negative* deflection of the difference wave (ERP to the target word “cheap” *minus* the ERP to the target word “expensive”) was observed in the trial with (AMV price ranges both for the iPhone (range 5) and Nokia phone (ranges 3, 4, and 5). We found no significant N400 deflection for the price range that matched the RMV for the iPhone (range 4) and Nokia mobile phone (range 2).

The time window used in the statistical analysis is depicted by a shaded gray rectangle. The significant differences (ERP to the target word “cheap” minus the ERP to the target word “expensive”) are indicated by shaded red (positive) and shaded blue (negative) rectangles (paired permutation test, *p* < 0.05). Price range: BVM—below market value; RMV—real market value; AMV—above market value.

### EEG Experiment 2

**Figure 4:**
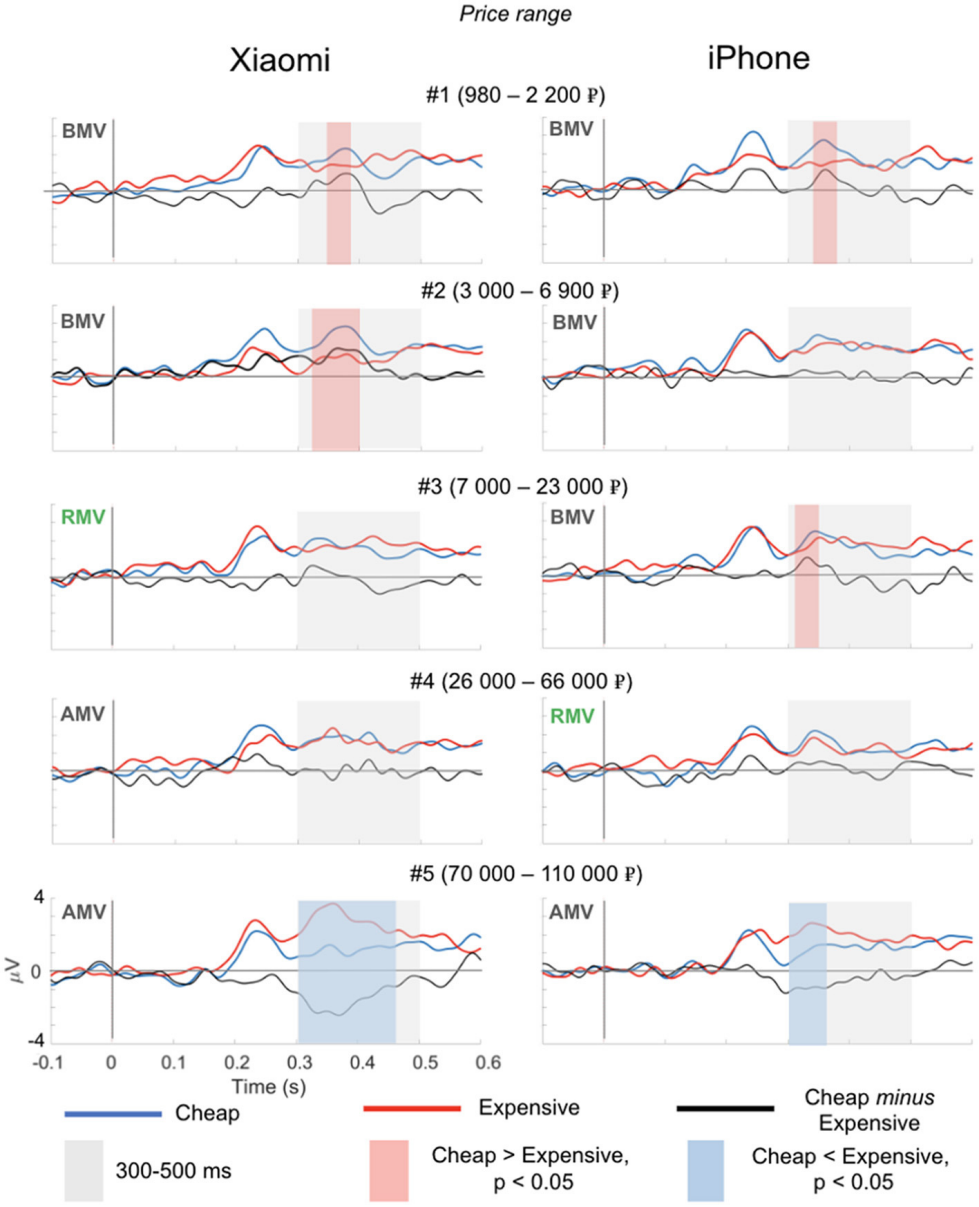
Event-related potentials to the target words “cheap” (blue curve) and “expensive” (red curve) in EEG Experiment 2. Across the price ranges, the N400 component reversed polarity in price ranges #3 and #4 in the trials containing photos of the Xiaomi mobile phone and price range #4 in the trials containing photos of the iPhone.

In the second experiment, we utilized the same experimental setup as in the first EEG experiment but exchanged the Nokia mobile phone with the more expensive Xiaomi smartphone. Importantly, we also observed the N400 magnitude change according to the price range. Similar to the first study, the magnitude of the N400 response gradually changed with the price range (Figure 4). The pattern of the N400 in the trials with the iPhone was identical to the results of Experiment 1 with a minor difference. Similarly, we observed a significant *positive* deflection in the difference wave (ERP to the target word “cheap” *minus* the ERP to target word “expensive”) in the trial with the BMV price range (ranges 1 and 3). However, in the trials with price range 2, the *positive* deflection was insignificant. Similar to EEG Experiment 1, for the iPhone, we found no significant N400 deflection for the price range that matched the RMV (range 4), while we observed a significant negative N400 deflection in the trials with the AMV price range (range 5). For the Xiaomi mobile phone, the inversion of the polarity of the N400 component was observed in the larger price range (range 4) compared to the iPhone (range 3). In the trial with the BMV price range (ranges 1 and 2), we observed a significant *positive* deflection in the difference wave, no significant N400 deflection for two price ranges at approximately the RMV (range 3 and 4) and a significant *negative* N400 deflection in the trials with the AMV price range (range 5).

The analyzed time windows are highlighted with gray rectangles. The significant differences (ERP to the target word “cheap” minus the ERP to the target word “expensive”) are depicted by shaded red (positive) and shaded blue (negative) rectangles (*p* < 0.05). Price range: BMV—below market value; RMV—real market value; AMV—above market value.

### MEG experiment

#### Evoked responses in the sensor space

##### The price judgment task

Importantly, to collect more trials and compare the price-related M400 with the standard semantic M400, in this version of the price judgment task we used only marginal price ranges 1 and 5). Therefore, we labeled evoked responses to the target word “cheap” following price range 1 and evoked responses to the target word “expensive” following price range 5 as the *congruent trials*. Similarly, we labeled the evoked responses to the target word “cheap” following price range 5 and evoked responses to the target word “expensive” following price range 1 as the *incongruent* trials.

**Figure 5:**
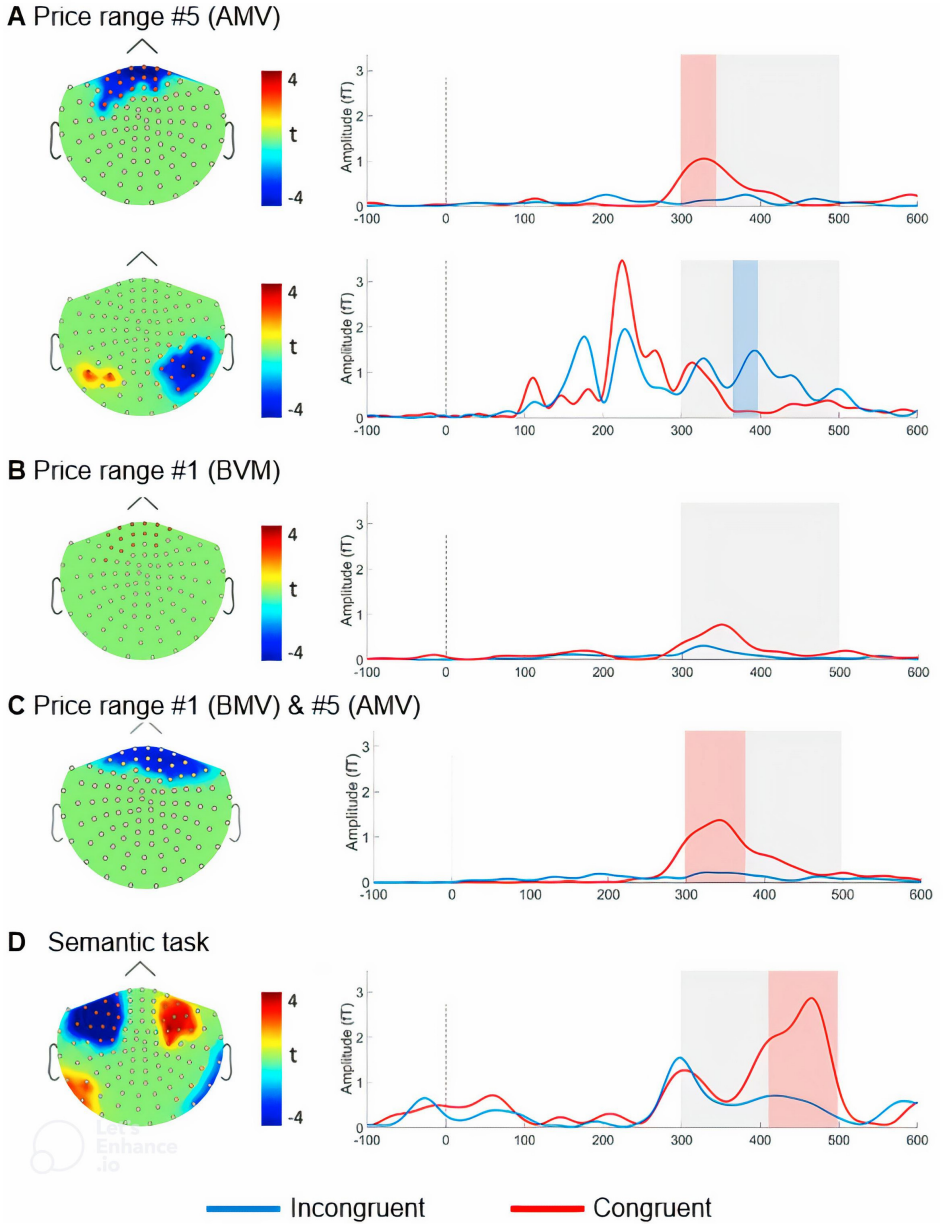
Results of MEG Experiment 3 (sensor space). Evoked responses to the *congruent* and *incongruent* target words in the price judgment task **(A–C)** and the semantic task **(D)** in the sensor space. Evoked responses to the target words following **(A)** relatively high (AMV) prices, **(B)** relatively low (BMV) prices, or **(C)** all price ranges (aggregated across *congruent* and *incongruent* conditions).

Figure 5 illustrates the spatial distribution of the t-scores resulting from the permutation test to compare the electromagnetic field responses to the *incongruent* and *congruent* target words in the price judgment task. We found a significant frontal difference between brain responses to *incongruent* target words and those to *congruent* target words in the 300–340 ms time window for trials with the AMV price range (Figure 5A, top). The permutation test revealed additional “opposite” parietal brain activity in the 376–396 ms time window on both magnetometers (Figure 5A, bottom) and gradiometers that can barely be associated with the semantic M400 component (Figure 5D) because the *congruent* target words caused a significantly greater response than the *incongruent* target words.

For the BMV range trials, no significant differences between evoked responses to *congruent* target words and those to *incongruent* target words were discovered (Figure 5B). When we aggregated evoked responses to *congruent* and *incongruent* target words for AMV and BMV price ranges, we observed a significant frontal difference between responses to *incongruent* target words and those to *congruent* target words in the time span of 300–350 ms (Figure 5C).

On the left: the scalp distributions of the t-scores obtained from the results of the FDR-corrected permutation test, which compares the evoked electromagnetic field to *incongruent* and *congruent* target/critical words in the magnetometer space (*p* < 0.05, FDR-corrected). The sensors for which the difference between *incongruent* target/critical words and *congruent* target/critical words was statistically significant are marked in red. The evoked electromagnetic fields were averaged across these sensors and depicted on the right side of the graph.

On the right: the evoked electromagnetic fields (global field power) were averaged across selected magnetometers that are marked in red on the left side of the figure. The analyzed time windows are highlighted with gray rectangles. The significant differences between *incongruent* target/critical words and *congruent* target/critical words are denoted by the shaded red (positive) rectangle and shaded blue (negative) rectangle, respectively (*p* < 0.05).

##### Semantic N400/M400 task

Using the standard N400 paradigm, we registered the MEG equivalent of the N400—M400. The FDR-corrected permutation test revealed that in the semantic task, the presentation of the *incongruent* critical word evoked a significantly (*p* < 0.05, FDR-corrected) larger M400 response than the *congruent* critical words in the time window of 414–498 ms after the stimulus onset (Figure 5D). The observed M400 component manifested both on magnetometers and gradiometers, covering wide areas on the sensors in the frontal sites (*p* < 0.05, FDR-corrected).

#### ERFs in the source space

##### Price judgment task

**Figure 6:**
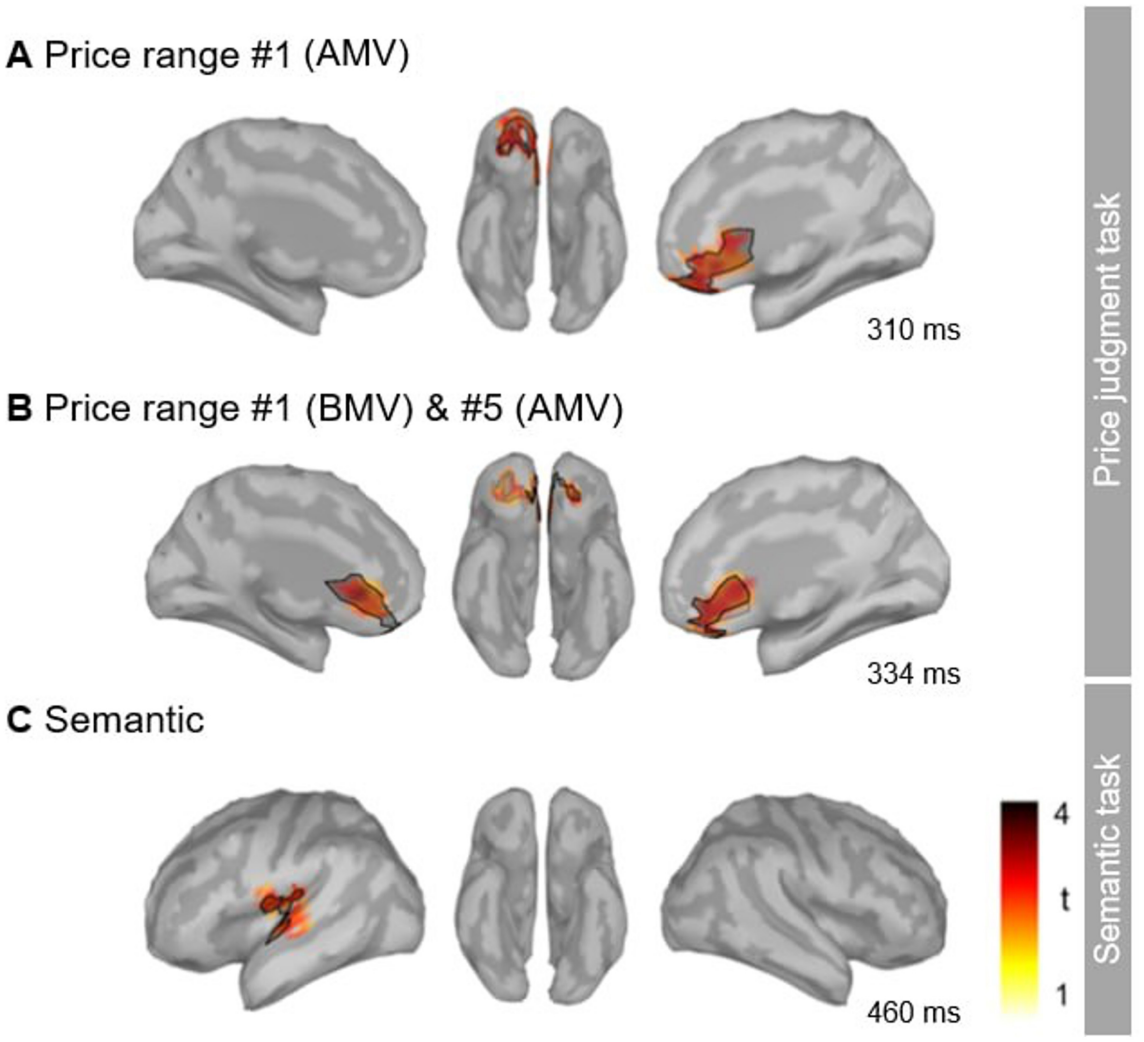
Source-localized MEG activity for the price judgment task **(A, B)** and the standard (N400/M400) semantic tasks **(C)**. Results of the cluster-corrected permutation tests depict the statistically significant difference between the evoked magnetic fields to *incongruent* target words and those to congruent stimuli in the source space (the black line outlines the spatial distribution of the largest clusters, *p* < 0.05, uncorrected).

When all price ranges were aggregated, the permutation test revealed significant clusters of activity in response to *incongruent* target words compared to *congruent* target words that spread over the right and left mPFC/ACC region within the 316–380 ms time window (Figure 6 and Table 2). For the trials with the AMV price range, two significant clusters at the right ventromedial PFC (vmPFC)/anterior cingulate cortex (ACC) were observed in the 300–350 ms time window (Figure 6 and Table 1). For trials with the BMV price range, no significant clusters were observed. Overall, activity related to the incongruent price was located at the vmPFC.

**Table 2:** Cortical MEG activity evoked by the incongruent target words in the price judgment task and the standard (N400/M400) semantic task.

**Price ranges**	**Hemisphere**	**Time window, ms**	**Cluster mass**	**Sign**	***p*-value**	**Brain structures**
**The price judgment task**						
AMV	Right	320–346	1,320	Positive	**0.01**	vMPFC/OFC, ACC
	Right	300–312	694	Positive	**0.044**	vMPFC/OFC, ACC
	Left	324–352	382	Positive	0.15	ACC
	Left	300–312	366	Positive	0.17	ACC
	Right	410–430	168	Positive	0.63	ACC
AMV and BMV	Right	320–346	1,444	Positive	**0.004**	vMPFC/OFC, ACC
	Left	316–354	881	Positive	**0.02**	vMPFC/OFC, ACC
	Right	348–380	778	Positive	**0.036**	vMPFC/OFC, ACC
**The semantic task**						
	Left	450–488	867	Positive	**0.006**	STG, ventral postcentral gyrus
	Left	410–434	751	Positive	**0.01**	STG, ventral postcentral gyrus
	Left	472–488	118	Positive	0.98	Superior frontal gyrus

Summary of the cluster statistics in the source space (MEG Experiment 3).

The significant clusters are indicated in bold typeface. BMV, below market value; RMV, real market value; AMV, above market value price ranges.

##### Semantic task

Finally, we localized the source of the standard M400 response to unexpected/*incongruent* words. In the semantic task, the cluster-corrected permutation test revealed two significant clusters of activity in the time window of 410–488 ms (Table 1). The cluster was distributed mostly over the left superior temporal gyrus (STG; Figure 6).

## Discussion

In the present study, which comprises three separate experiments, we investigated the brain mechanisms of the visual processing of the non-optimal price. We focused on the N400 component: a deflection of the difference wave (ERP to the target word “cheap” minus the ERP to the target word “expensive”) within the 300–500 ms time-window. In two EEG experiments and one MEG experiment, we found evidence in support of our hypothesis that the N400 component is sensitive to the price-product association. Consistent with our hypothesis, within 300–500 ms after the onset of the incongruent target words that followed the price that substantially differed from the market price (RMV), we observed the strongest N400 compared to the target words that followed prices that were close to the market price. Importantly, for all products including the cheap Nokia model, middle-range Xiaomi model and expensive iPhone, across all price ranges, the N400 flipped polarity in the trials containing the prices that were close to the market price (RMV). Overall, the N400 was the largest in response to the target word “expensive” in the trials with the lowest price (BMV) ranges and in response to the target word “cheap” in the trials with the highest price range (AMV).

First, in the case of the Nokia mobile phone, the N400 (differential wave) inverted its polarity in trials with the price varying in the range of 3,000–7,000 monetary units (price range 2). Second, in the case of the more expensive iPhone, N400 inversion was observed in trials with a much higher price varying from 26,000 to 66,000 monetary units (range 4). Last, for the Xiaomi mobile phone, we observed the N400 polarity inversion within a wider price range of 7,000–66,000 monetary units (ranges 3–4). The broader price range corresponding to the polarity inversion of the N400 for the Xiaomi mobile phone can be related to a relative novelty of the brand for the local market in the year in which the experiment was run. One can speculate that for the lesser-known brand, the relatively broad price range delineating the N400 polarity inversion may reflect a weak price–product association leading to uncertainty in the price positioning. Importantly, the N400 dynamics showed a high reproducibility, since our N400 results were replicated in both EEG experiments with the iPhone.

The mismatch of the target words “cheap” or “expensive” with the price of the mobile phone evoked a centroparietal N400 response in the 300–400-ms time window, which is ~100 ms earlier than the latency of the standard semantic N400 (Lau et al., 2008). The centroparietal localization of the response was typical for the semantic N400 (Kutas and Federmeier, 2011). Nevertheless, as shown in many studies, several neural networks can be involved in such distributed centroparietal activity and only fMRI or MEG recordings can lead to more precise N400 source localization, as discussed in the review by Lau et al. (2008).

In MEG Experiment 3, we observed significant M400 responses to the incongruent target words following prices that were too low or too high. To make a more robust statistical analysis, we also aggregated BMV and AMV price ranges and observed M400 responses in the bilateral vmPFC and ACC regions within the 300–380 ms time window. Therefore, our findings suggest that the vmPFC and ACC play a crucial role in monitoring the congruence between product attributes and their prices. This cognitive process is a key component of the brain's valuation system, as it helps determine whether a product's price aligns with its perceived value. Various studies show that the vmPFC forms the core of a “valuation system” (Kable and Glimcher, 2009).

An extensive meta-analysis by Bartra et al. (2013) demonstrated that the vmPFC consistently exhibits activity related to subjective value during both decision-making and outcome delivery (when the consequences of a decision are presented to the individual). This pattern holds true for decisions involving monetary rewards as well as primary rewards, such as food or social interactions. Additionally, Basten et al. (2010) discovered that the vmPFC plays a crucial role in merging neural signals associated with both benefits and costs. Specifically, the vmPFC integrates these benefit and cost signals into a single, unified representation that reflects the subjective value of a decision. Some studies have also shown that the vmPFC plays a key function in brand preferences and price-based decision-making (Bush et al., 2002; Koenigs and Tranel, 2008; Paulus and Frank, 2003; Plassmann et al., 2007; Wallis and Miller, 2003). For example, Plassmann et al. (2008) reported that during blind testing, brain activity in the vmPFC was stronger in trials when the participants believed that wine was expensive compared to the trials in which participants believed that the same wine was cheap. The vmPFC activity also correlated with actual preferences for wine among the participants. These pioneering results suggested that the instantaneous experience of pleasure from a product is influenced by pricing and that this effect could be mediated by the vmPFC (Plassmann et al., 2007).

According to the current state of knowledge, the ACC—adjacent to the vmPFC—is involved in reward-based learning and decision-making. Bush et al. (2002) identified the ACC as a region that integrates cognitive and emotional influences, enabling the evaluation of action outcomes in terms of rewards or errors. Specifically, their findings suggest that the ACC contributes to adaptive behavior by dynamically monitoring performance and signaling when adjustments are needed. Similarly, Vecchiato et al. (2011) emphasized the ACC's involvement in reward processing within the context of consumer neuroscience, highlighting its role in evaluating preferences and guiding choices.

Altogether, our complex MEG/EEG results demonstrate that the brain source and timing of the price-related M400 response differed from those of the standard M400 response evoked by the semantically incongruent words. In the classical semantic task (MEG Experiment 3), incongruent target words evoked stronger brain activity in the later 410–488 ms time window within the left STG and ventral postcentral gyrus. This localization is consistent with the dominant model of semantic processing during language comprehension (Lau et al., 2008). These findings partly support the results of an earlier MEG study by Halgren et al. (2002), who demonstrated an M400 response to the incongruent sentence ending within a 300–500 ms time window localized in the left hemisphere in the anterior temporal, perisylvian, orbital, frontopolar, and dorsolateral PFCs. Thus, our MEG findings, on one hand, replicate the previous studies of the standard semantic M400, but on the other hand, demonstrate that the sources and timing of the price-related M400 response differ from those of the classic semantic M400.

Interestingly, we found the statistically significant price-related M400 only in the trials with the highest price ranges (AMV). A possible explanation is that the neural correlates of the price-related semantic violations could have distinct brain sources in the contexts of high and low prices. In contrast to EEG method, MEG is less sensitive to the radial orientation of neuronal currents. Thus, if in the contexts of high and low prices, price-related M400-like responses are generated by neural currents with different orientations, the MEG would reveal only tangential sources. Alternatively, the absence of the M400 in the trials with low-prices can be explained by their extreme unrealistic deviation in price range 1 from the market price of the iPhone. The highest price range 5, was 3–4 times higher than the market price, while the lowest price range 1, was 10–100 times smaller than the market price, making price range 1 quite implausible. For example, Lee (2019) demonstrated that the implausibly high discounts led to a lower perception of a deal, including perceived savings, price fairness and perceived value. Additionally, participants could focus more on the high-price ranges, largely disregarding lower prices (Shirai, 2015). However, alternative interpretations are highly improbable since both trials with the lowest price range and trials with the highest price range evoked robust N400 in two EEG experiments.

The current study has several limitations that must be acknowledged and considered for future research. For example, we used unequally spaced price ranges to seamlessly cover a wide range of prices and to compare mobile phones of obviously distinct market values. The linear spacing of prices would dramatically increase the duration of the experiment, making it highly uncomfortable for participants. Future studies should test different fine-grained price ranges. In addition, questionnaires that include measures of personality, personal income, and brand awareness could be utilized in future research.

## Conclusion

Overall, our results confirmed and extended the results from previous studies of the semantic N/M400 by localizing the classic M400 at the left STG and ventral postcentral gyrus within the 410–488 ms time window. We also reported N400-like evoked brain activity in response to non-optimal prices that was localized in the vmPFC and ACC. Thus, our results indicate that neural signatures of the price-related semantic violations largely differ from the neural signatures of lexical and semantic anomalies. Our results also highlight the neural mechanisms of the semantic representation of price–product associations. Our results could be used in neurotechnologies to estimate the optimal price for new products or new brands. The N400-based methodology could evolve into a simple and robust tool for testing the price positioning of products.

## Data Availability

The datasets presented in this study can be found in online repositories. The names of the repository/repositories and accession number(s) can be found below: https://disk.yandex.ru/d/IFMh4jFL4jOecw.
