# Supplementary material for: Neural correlates of the non-optimal price: an MEG/EEG study
Source: Front Hum Neurosci. 2025 Jan 28;19:1470662. doi: 10.3389/fnhum.2025.1470662 (PMC11811784; doi:10.3389/fnhum.2025.1470662)
Supplement: Supplementary file 1 [file Data_Sheet_1.docx]

Neural correlates of the non-optimal price: an MEG/EEG study

**Supplementary materials**

**Table S1.**

Price ranges, number of trials and values of within-range price grading.

| Range | Price range (RUB) | Number of trials | Increment (RUB) |
| --- | --- | --- | --- |
| *EEG experiments. Stimuli: iPhone XS, Nokia 105 S, Xiaomi Mi A2* | | | |
| **1st** | 980 – 2200 | 40 | 30 |
| **2nd** | 3 000 – 7 000 | 40 | 100 |
| **3d** | 7 400 – 23 000 | 40 | 400 |
| **4th** | 26 000 – 66 000 | 40 | 1 000 |
| **5th** | 67 000 – 110 000 | 40 | 1 000 |
| *MEG experiment 3. Stimulus: iPhone XS* | | | |
| **1** | 500 – 7000 | 40 | ~150 |
| **2 (Filler)** | 15 000 – 40 000 | 4 | ~6 000 |
| **3 (Filler)** | 50 000 – 120 000 | 8 | ~9 000 |
| **4 (Filler)** | 140 000 – 180 000 | 4 | ~10 000 |
| **5** | 210 000 – 310 000 | 40 | ~2 500 |
| *MEG experiment. Stimulus #2: Nokia 105 S* **- Filler** | | | |
| **1** | 0 – 200 | 4 | ~50 |
| **2** | 300 – 500 | 4 | ~50 |
| **3** | 650 – 1 500 | 4 | ~225 |
| **4** | 2 500 – 4 500 | 4 | ~500 |
| **5** | 5 000 – 10 000 | 4 | ~1 250 |


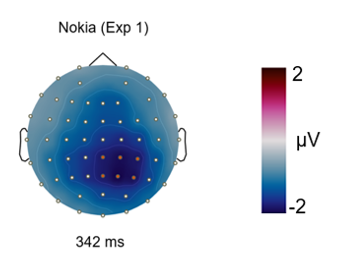


Figure S1. Spatial distribution of the maximal N400 magnitude. The electrodes used to form the cluster marked in red.
